# New Artificial Urinary Sphincter Devices in the Treatment of Male Iatrogenic Incontinence

**DOI:** 10.1155/2012/439372

**Published:** 2012-04-11

**Authors:** Ioannis Vakalopoulos, Spyridon Kampantais, Leonidas Laskaridis, Vasileios Chachopoulos, Michail Koptsis, Chrysovalantis Toutziaris

**Affiliations:** First Department of Urology, Aristotle University of Thessaloniki, Thessaloniki 54635, Greece

## Abstract

Severe persistent stress incontinence following radical prostatectomy for prostate cancer treatment, although not very common, remains the most annoying complication affecting patient's quality of life, despite good surgical oncological results. When severe incontinence persists after the first postoperative year and conservative treatment has been failed, surgical treatment has to be considered. In these cases it is generally accepted that artificial urinary sphincter is the gold standard treatment. AUS 800 by American Medical Systems has been successfully used for more than 35 years. Recently three more sphincter devices, the Flow-Secure, the Periurethral Constrictor, and the ZSI 375, have been developed and presented in the market. A novel type of artificial urinary sphincter, the Tape Mechanical Occlusive Device, has been inserted in live canines as well as in human cadavers. These new sphincter devices are discussed in this paper focusing on safety and clinical results.

## 1. Introduction

In recent years, despite improvement in the surgical technique, the prevalence of Postprostatectomy Urinary Incontinence (PPUI) has increased due to rising number of radical prostatectomies performed annually [1]. Iatrogenic-induced sphincter incompetence is the reason of postoperative stress incontinence in 95% of cases. The reported PPUI rates vary from 5% to 48%. This large variation may be attributed mainly on the influence of the interviewing physician and a lack of a standardized definition of “post prostatectomy incontinence” [2]. 

 Noninvasive therapy and particular pelvic floor muscle training is the first-line treatment for early incontinence following prostatectomy within the first 6 to 12 months [2]. Lifestyle interventions and pharmacotherapy (duloxetine) are also recruited in this attempt [3, 4]. Despite this conservative intervention, up to 10% of patients with PPUI exhibit a persistent and moderate-to-severe incontinence for more than one year postoperatively [5]. For these patients surgical treatment is recommended [2].

The Artificial Urinary Sphincter (AUS) 800 (American Medical Systems, Minnetonka, MN, USA), despite the new surgical treatment options (slings, injection of bulking agents, stem-cell therapy), remains the gold standard for persistent moderate-to-severe stress urinary incontinence due to Intrinsic Sphincter Deficiency (ISD) [1, 2, 6]. In effort to keep the good success rates and improve some disadvantages of AUS 800 (high cost, complications, and relative difficult insertion), four new devices have been developed in recent years [1]. We attempt to present technical characteristics and insertion procedures for these devices and to report safety and efficacy data, where they are available.

## 2. FlowSecure TM (RBM-Med)

The FlowSecure artificial urinary sphincter is a new prosthesis for the management of urinary incontinence due to ISD that has been designed and developed by Professors Craggs M. D. and Mundy A. R. at London's Institute of Urology and Nephrology, in 2006 [7]. Unlike AUS 800, this sphincter is an adjustable prosthesis filled with normal saline without contrast. Plain X-rays cannot therefore be used for monitoring, and ultrasound scan is the adequate radiographic technique for evaluation. Except verifying prosthesis status, ultrasound also allows measuring of the postvoid residual volume and calculation of the urethral closing pressure [8]. Moreover, MRI can ensure the precise position and integrity of all components of the sphincter [9]. 

**Figure 1:**
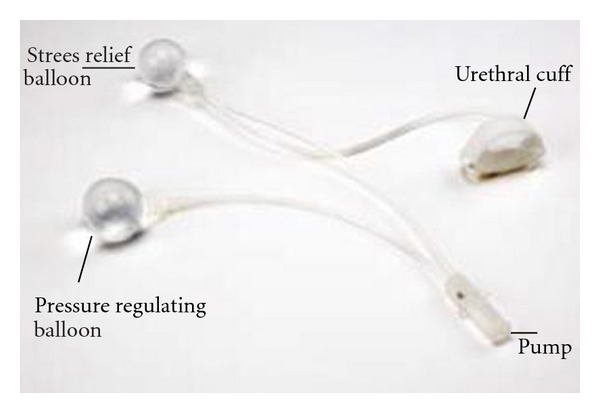
FlowSecure artificial urinary sphincter.

The FlowSecure sphincter is a one-piece device consisting of two reservoirs placed in the paravesical space, a cuff that surrounds the urethra and a control pump with a self-sealant port that is placed in patient's scrotum (Figure 1). The first reservoir regulates resting urethral pressure and the other relieves stress pressure during intra-abdominal increase. During bladder filling the cuff connected with the pressure regulating reservoir compresses and keeps the bulbar urethra closed at low pressure. When intra-abdominal pressure rises, the stress relief balloon provides additional pressure to the cuff to maintain continence. The fluid pressure of the prosthesis may be regulated by injecting or removing saline through the self-sealing port in the control pump located in patient's scrotum [10]. When the patient wishes to void he only has to press the control pump until a good urine flow is achieved. In this way the cuff is emptied by moving the fluid from it to the pressure-regulating reservoir. Redirection of fluid flow and filling of the cuff is recovered when compression on the pump stops [11].

Indications for implantation of the FlowSecure device in order of significance are postprostatectomy urinary incontinence, incontinence due to congenital abnormalities, neurogenic bladder with ISD, and women stress incontinence, where other surgical procedures have failed [9–12].

Both perineal and suprapubic access are needed for prosthesis implantation. Pressure-regulating and stress relief reservoirs are lodged in Retzius space through the suprapubic incision. The cuff is placed through the perineal incision around the bulbar urethra as it is designed to transmit direct pressure over the urethra. By blunt dissection a space is created between the two incisions to pass the tubing, as well as a subcutaneous space in the scrotum where control pump is placed. FlowSecure is accompanied by a plastic trocar and its obturator, which allows transposition of urethral cuff between Retzius space and perineum, and a tube of glue for temporary fixation of the belt over the cuff when adjusting it [11]. The control pump should be used as soon as the scrotal edema disappears. In case of persistent urinary retention, patients must be taught to perform intermittent self-catheterizations until the problem resolves. It is important that, during the catheterizations period, the patient must use the control pump to empty the cuff, even if he cannot void. The patient should be reevaluated for continence status 2 to 4 weeks after discharge. If he maintains continence, the prosthesis does not need pressurization. In those patients, who do not regain continence, pressurization of the system must be carried out. Under strict aseptic conditions, local anesthesia is administered at the scrotal area where the control pump is located. Some saline is injected through the self-sealing port using an orange 25 G 15 mm needle and a 10 mL syringe. The needle must be inserted longitudinal to the pump, to avoid damaging the device. The prosthesis pressure is directly dependant on the injected volume, following a pressure/volume curve. Ideally, the first pressurization reaches between 40 and 50 cm H_2_O, which normally takes about 4 to 6 mL of saline. The patient is advised to be re-evaluated two weeks after initial pressurization. At this time, fluid can be added or removed from the system to accommodate to the patient's needs. It is not advisable, during subsequent pressurization, to add more than 2 mL of saline per session [9]. Proper device function must be monitored by free uroflowmetry, ultrasound scan, and clinical history [8].

Knight et al. presented 9 male patients (mean age 66 years) with urodynamically proven stress incontinence due to radical prostatectomy treated with implantation of FlowSecure sphincter. The patients were followed for a minimum period of 12 months. All 9 patients recovered well from surgery. Two devices had to be removed for technical reasons. The mean leakage for the remaining 7 patients prior to implantation was 771 ± 658 mL corresponding to a continence index of 54%. Twelve months later the leakage had statistically significantly reduced to 52 ± 36 mL (*P* < 0.05) and the continence index increased to 97%. There was no significant difference in bladder capacity or flow rate. Four patients required additional pressurization to achieve optimal continence and this was carried out without complication [13]. In another study by Rodriguez et al. 100 patients with stress urinary incontinence of various etiologies underwent bulbar urethra (96%) or bladder neck (4%) implantation of a FlowSecure device. All patients had tried conservative treatments and also 59 patients had undergone unsuccessful surgical procedures (suburethral slings, bulking agents, Proact, and AUS-800). Nine patients had undergone previous pelvic radiotherapy. At implantation the sphincters' pressure was left at atmospheric level in all cases. Patients attended for initial pressurization 2–4 weeks postoperatively and were recalled at two-week periods for evaluation and repeat pressurization, if it was required. Overall, 3 pressurizations procedures were required to achieve socially satisfactory continence in 89 patients. The implanting procedure lasted in average 38–47 minutes. Mean inpatient stay was 4.3 days. 53 patients had postoperative self-limited scrotal hematoma. Implants had to be removed in 28 patients (28%) due to early infection (8%), late infection secondary to pressurization procedures (5%), perforation of the pump at pressurization (9%), and mechanical failure (6%). No erosions were noted [14].

The main advantage of FlowSecure device over AUS 800 is that while the later exerts a high constant pressure over the urethra, the former increases pressure on the urethra instantly, only during stress increase of intra-abdominal pressure. During the deactivated position the cuff turns back to the initial low pressure not exceeding 40 cm H2O, thus minimizing danger for urethral erosion [11].

In conclusion, FlowSecure artificial urinary sphincter is easy to implant, with low risk of mechanical failure, and adjustable to the patient's continence needs. Its main purpose is to achieve total patient continence during periods of raised abdominal pressure while subjecting urethra to the lowest possible pressure during resting. Urethral ischemia potentially leading to atrophy and erosion is thus prevented [8]. Use of one-piece prosthesis also decreases the risk of infection due to intraoperative handling and minimizes the chance of mechanical failure derived from errors during assembly [13]. All these may predict a promising future. However, more time and studies will be needed to define the role of this sphincter in the management of stress incontinence resistant to other treatments.

## 3. Periurethral Constrictor (Silimed, Rio de Janeiro, Brazil)

**Figure 2:**
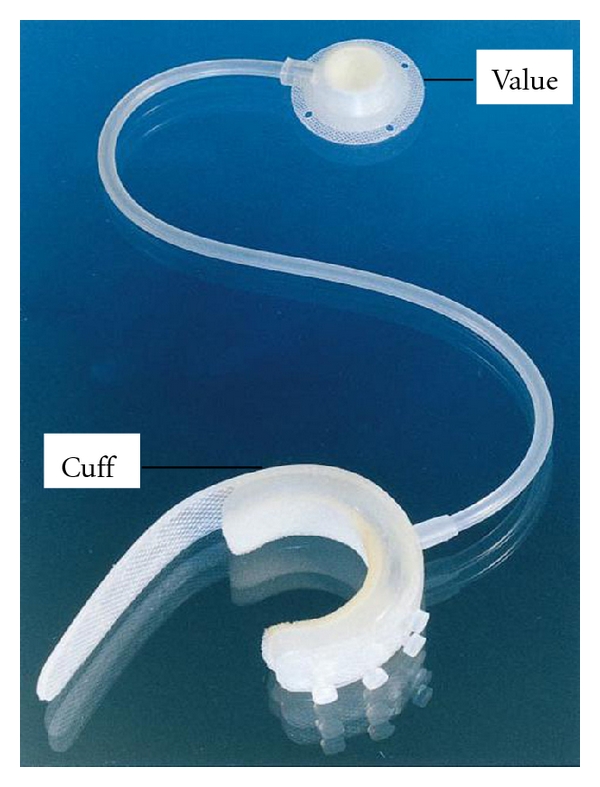
The Periurethral Constrictor.

The Periurethral Constrictor (PUC) was developed by Dr. Fabio Vilar in 1996. It was designed for implantation in pediatric patients to treat deficient bladder sphincter function [15]. The PUC is a one-piece, two-part device. It is comprised of a constrictor cuff linked by a 20 cm silicone tube to a valve, which is elliptical in shape and rounded at the edges (Figure 2) [16]. The adjustable cuff is implanted around the bladder neck through suprapubic approach or bulbous urethra through perineal incision [17]. The valve is placed in a space accessible by percutaneous puncture, usually in the subcutaneous space between the umbilicus and the iliac crest. The injection port is designed to accommodate a fine Huber needle [18].

The system works hydraulically by the injection of sterile saline solution through the self-sealing valve in order to promote a static occlusive pressure on the cuff. The activation of the PUC takes place 6–8 weeks after implantation surgery. This requires filling the bladder and then injecting further saline into the system for as long as the occlusive pressure obtained allows good urine flow without significant increase of the post void residual urine volume [16].

A limited number of studies with controversial results have been published for using PUC in PPUI, excluding studies focused in its use in pediatric population, especially for the treatment of neurogenic urinary incontinence due to ISD [15, 18, 19]. In a study performed by Simone et al. 43 patients with mild urinary incontinence following radical prostatectomy were treated by PUC implantation. Overall successful rate was 86%. Postoperative complications occurred in 6 patients (1 hematoma, 1 erosion, 2 infections, and 2 malfunctioning devices). Only the first two complications were managed by device removal [20]. Schiavini et al. retrospectively studied 30 patients with PPUI and PUC implantation for a mean period of 42.1 months. At the time of implantation the reported mean use of pads was 4.4 per day. In 22 patients (73.3%) the devices were functional leading to a good continence result. In 7 patients the device was removed because of cuff erosion (4 patients, 13.3%) and infection (3 patients, 10%). An eighth patient remained incontinent after the device reactivation because of detrusor hyper-reflexia [16]. On the other hand, Lima et al. presented a study with 82.2-month mean followup which reported a very high device removal rate 41.07% [17]. The average time between surgery and the removal of the device was 22.6 months. The most frequent complication was urethral erosion in 15 patients (26.78%). Comparing erosion rates of AUS 800, ranging from 1.7% to 4.5%, the present study presented higher rates [21]. Other complications were mechanical malfunction in 5 (8.9%), urethral stenosis in 3 (5.3%), urinary fistula in 2 (3.5%), infection in 2 (3.5%), and persistent urinary tract infection in 1 case (1.7%). In patients in whom the device was not removed (33), only 17 from them were continent, representing an overall success rate of 30.35% [17].

The above results, suggest that further studies are required to determine the safety and efficacy of this type of artificial urinary sphincter. However, simplicity and low cost of PUC are important characteristics in its favor comparing with AUS 800 [16]. The one-piece design makes the device easier to implant and the absence of connections reduces the chances for leakage and kinks [19]. The characteristics of the cuff allow spontaneous voiding and catheterization without the need of previous emptying the cuff. It can be deactivated, under activated or reactivated at any time by simply punctuating the subcutaneous port to add or subtract fluid [18]. Based on these observations, more studies are needed to bring safer conclusions.

## 4. ZSI 375 (ZEPHYR Surgical Implants, Swiss-French)

**Figure 3:**
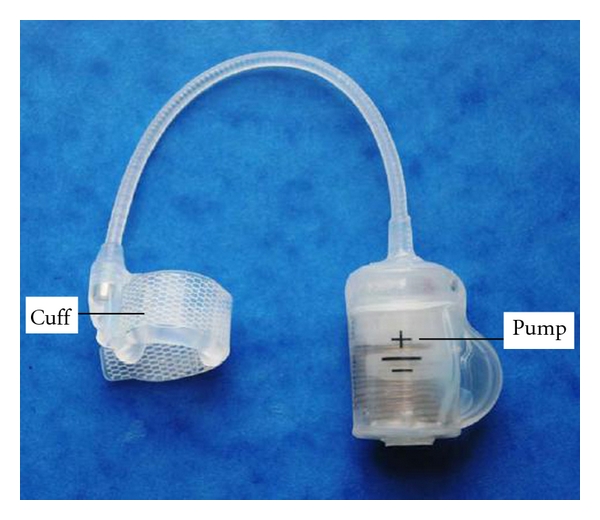
The ZSI 375.

ZSI 375 is an artificial urinary sphincter produced by ZEPHYR Surgical Implants, a Swiss-French company. The system was designed and created by Dr. Christophe Gomez-Llorens and Raphael Gomez-Llorens in 2005 [22]. It is used to treat severe urinary incontinence due to ISD. The ZSI 375 is a one-piece medical device that can be implanted only in men. It is made by silicone elastomer and filled with sterile normal saline solution. It consists of an inflatable and adjustable cuff that fits around the urethra and a pump with an embedded pressure-regulating tank placed in the scrotum connected with the cuff by a 110 mm silicone tube (Figure 3). The maximal pressure in the cuff must not exceed 350 mbar. The ZSI 375 is filled by normal saline solution. There are two compartments in the device: a hydraulic circuit and a compensation pouch circuit separated by a piston. Spontaneously, the spring pushes the piston up and the piston pushes the saline solution of the hydraulic circuit into the cuff. The pressure in the hydraulic circuit is not the only factor of the cuff efficiency. To obtain a good continence result, the deflated cuff must compress the urethra. The ZSI 375 has the advantage to increase the issued pressure and give the chance to readjust the cuff [22].

Two incisions are needed for the implantation of the device. Through a perineal incision the cuff is placed around the bulbar urethra. The pump unit is placed in a subdartos pouch through an inguinal incision. Six to eight weeks later the device is activated by pressing an activation button. If necessary, it is possible to inject 1 or 2 mL of normal saline in the compensation pouch through the scrotum to increase the issued pressure [22].

The advantages of ZSI 375 over AUS 800 are reduced cost, the opportunity to adjust the issued pressure of the device, and the possibility to re-adjust the cuff in case of postoperative urethral atrophy [22].

In a study performed by Sandul et al. 34 men with urine incontinence after treatment for prostate cancer were treated by ZSI 375. Thirty-two of them had radical prostatectomy, six of those also had adjuvant radiotherapy, and two had brachytherapy followed by TURP to relieve outlet obstruction. Eight patients had already undergone a male perineal sling operation. Initial sphincter closure pressure was elected to be 60–70 cm H_2_O. 60% of the patients needed further increase of the pressure, something which was performed as an office procedure. The interval for primary activation ranged from 4 to 6 weeks. With a maximum followup of 20 months, no surgical revision was necessary for mechanical malfunction. Infection of the device occurred in 2 (5.8%) patients requiring device removal. Overall, social acceptable continence was achieved in 94.2% (32 patients) [23]. Llorens et al. studied 17 men, after 1 year of implantation of the ZSI 375. 14 patients were incontinent after radical prostatectomy and 3 patients after TURP. All patients had tried previous conservative treatments without success. Total continence was defined as dry, and social continence was defined as a minimal leakage requiring at the most one pad daily with activity. All other results were defined as incontinence. In14 patients the initial pressure adjusted to 60–70 cm H_2_O and in 3 patients to 70–80 cm H_2_O. Mean hospitalization duration was three days. Implantation and recovery were uneventful for 12 patients. Four patients in whom the pump unit was implanted through the perineal incision presented permanent scrotum edema making the manipulation of the pump difficult. This led to the reimplantation of the pump in a subdartos scrotal pouch through a scrotal incision. After re-implantation, one patient presented extrusion of the pump unit and the device had to be removed. Finally one patient presented infection leading to artificial sphincter removal five days after the procedure. None of the remaining 15 patients demonstrated bladder overactivity, chronic urinary retention, or any other adverse effect. According to the results the three patients implanted with 70–80 cm H_2_O issued pressure in the system were dry. For the 12 patients implanted with the 60–70 cm H_2_O issued pressure in the system, three became completely dry, three achieved social continence, and six were still incontinent using two to three pads per day. Eleven patients implanted with 60–70 cm H_2_O were initially satisfied with their continence results. However, after in-situ injection of one mL of saline solution in the compensation pouch, the issued pressure increased 10 cm H_2_O to 70–80 cm H_2_O and the patients improved or achieved social continence [22]. 70–80 cm H_2_O seems to be the most efficient issued pressure because the pressure-regulating system of ZSI 375 is not submitted to abdominal pressure.

The innovative features of the ZSI 375 are the following: it is a one-piece device thus facilitating preparation and implantation; it contains an adjustable cuff mounted in a curve to reduce creasing and fracture danger; it offers the possibility to increase the issued pressure of the device in situ achieving better continence results; finally, preparation and implantation of the ZSI 375 are technically simple and quick with no serious adverse events associated with the device [22].

## 5. The Tape Mechanical Occlusive Device (GT Urological, Minneapolis, MN)

A new type of artificial sphincter is being developed utilizing a spring-loaded mechanism for applying circumferential pressure in the urethra, which is easy to implant and simple to use. This artificial urinary sphincter is the Tape Mechanical Occlusive Device (TMOD) (GT Urological LLC, Minneapolis, MN), a one-piece device that is manually controlled by the patient through its ON/OFF buttons [24].

**Figure 4:**
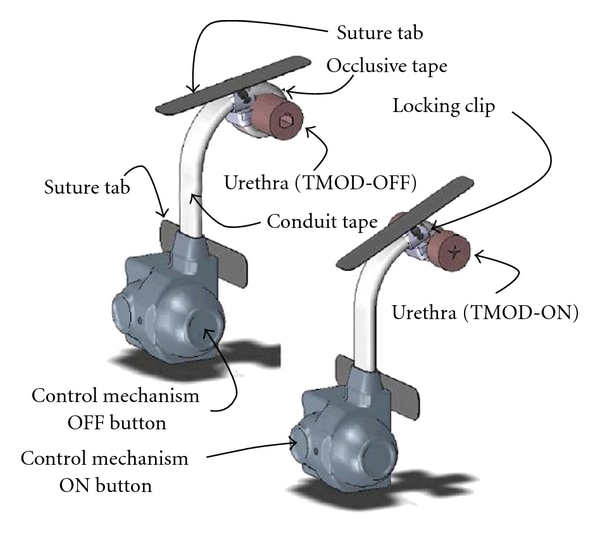
The Tape Mechanical Occlusive Device.

The TMOD is a totally implantable, one-piece artificial urinary sphincter (Figure 4) consisting of an occlusive tape and a conduit tape, connected to a control mechanism. The conduit tape originates at the control mechanism and the occlusive tape connects to the conduit tape. The conduit tape is of sufficient length to allow placement of the occlusive tape at the bulbous or penoscrotal urethra without creating undue tension on the conduit tape. The scrotally implanted control mechanism consists of a titanium casing, housing a three-metal alloy spring that applies tension to sutures running through the conduit and occlusive tapes. The control mechanism has ON and OFF buttons and is covered with a flexible silicone boot that prevents tissue in-growth. The boot has a port for injection of saline into the device that displaces air and creates an isotonic interior. This same port is designed for antibiotic flushing per surgeon discretion. In the ON position, the occlusive tape contracts and is designed to apply radial pressure to the urethra of 50–80 cm of water. The degree of radial pressure was chosen from clinical experience with the AUS 800 in order to limit urine leakage while minimizing urethral perfusion. There is a locking clip that locks the occlusive tape to itself to form an annular occlusive ring around the urethra. This can easily be unclipped for tape removal if repositioning or removal of the TMOD is required. Suture tabs are attached to both the control mechanism and occlusive tape to anchor the device in place and prevent migration [24].

In humans is anticipated to implant the occlusive tape in an open, deactivated condition, allowing it to remain in that condition for 6 weeks postoperatively to permit healing. The physician would then activate the device to apply pressure to the urethra by depressing the ON button through the intact scrotal skin. The patient can then depress the control mechanism OFF button to again remove occlusive pressure from the urethra and allow for unobstructed voiding or lock it in the deactivated position for voiding or nocturnal deactivation. To reactivate, the patient can push the ON button [24].

The TMOD has been implanted in canines to assess its functionality, occlusive efficiency, and biocompatibility and in human male cadavers to assess its occlusive efficiency and sizing, with encouraging results. Activation of the implanted TMOD resulted in intraurethral pressures within the desired range of 50–80 cm H_2_O. The device has proven to have no evidence of systemic toxicity. It has met the requirements for reliability and biocompatibility [24].

The TMOD seems to offer several advantages over the currently available AUS 800. It is a single piece device that does not require assembly reducing preparation time by operating room staff. It is nonhydraulic and requires no pressure-regulating balloon placement leading to less dissection. The reduced width of the tape minimizes the dissection around the bulbar urethra reflecting a lower risk of urethral injury during the operation. The simplicity of the ON/OFF button operation allows easier patient control. The TMOD has not been implanted in live human patients yet and human clinical trials should follow given the proof of technical feasibility, biocompatibility, and lack of systemic toxicity.

## 6. Conclusion

 The first artificial urinary sphincter was introduced in 1973 to treat ISD [25]. After its introduction the basic device design changed to the fifth-generation model, the AUS 800, in 1983 [26]. Despite its good success rate, in order to decrease mechanical failure, numerous changes have been made to the various components of this device; however, its basic design and mode of operation have remained unchanged for over 20 years. Recently, new devices have been developed to overcome the disadvantages of AUS 800. The controversial results in success and complication rates emphasize that these new devices need to be implanted in greater numbers of patients and with longer follow-up periods. If this experience reveals that one or more of them present potential advantages, these new artificial urinary sphincters will become an important tool in the management of men suffering from PPUI owing to ISD.

## References

[1] Bauer RM, Gozzi C, Hübner W (2011). Contemporary management of post prostatectomy incontinence. *European Urology*.

[2] Schröder A, Abrams P, Andersson KE, Arnheim AG (2010). Guidelines on urinary incontinence. *EAU Guidelines*.

[3] Goode PS, Burgio KL, Johnson II TM (2011). Behavioral therapy with or without biofeedback and pelvic floor electrical stimulation for persistent postprostatectomy incontinence: a randomized controlled trial. *JAMA*.

[4] Mariappan P, Alhasso A, Ballantyne Z, Grant A, N’Dow J (2007). Duloxetine, a serotonin and noradrenaline reuptake inhibitor (SNRI) for the treatment of stress urinary incontinence: a systematic review. *European Urology*.

[5] Penson DF, McLerran D, Feng Z (2005). 5-Year urinary and sexual outcomes after radical prostatectomy: results from the prostate cancer outcomes study. *Journal of Urology*.

[6] Herschorn S (2008). The artificial urinary sphincter is the treatment of choice for post-radical prostatectomy incontinence. *Journal of the Canadian Urological Association*.

[7] Craggs MD, Chaffey NJ, Mundy AR (1991). A preliminary report on a new hydraulic sphincter for controlling urinary incontinence. *Journal of Medical Engineering and Technology*.

[8] Rodríguez DA, Barranco LF, García-Montes F, Salvá AM, Moragues MO (2009). Follow-up of the FlowSecure artificial urinary sphincter. *Actas Urologicas Espanolas*.

[9] García Montes F, Vicens Vicens A, Ozonas Moragues M (2007). ‘FlowSecure’ artificial urinary sphincter: a new adjustable artificial urinary sphincter concept with conditional occlusion for stress urinary incontinence. *Actas Urologicas Espanolas*.

[10] García-Montes F (2009). FlowSecure*™* artificial urinary sphincter for the treatment of stress urinary incontinence after radical prostatectomy. *Archivos Espanoles de Urologia*.

[11] Vallejo JEB, Montes FG, Rosell LC, Maresma CM, Robles JM (2009). Implantation technique of the artificial urinary sphincter flow secure*™* in the bulbar urethra. *Archivos Espanoles de Urologia*.

[12] García-Montes F, Vicens-Vicens A, Ozonas-Moragues M, Oleza-Simo J, Mora-Salvá A (2007). Surgical implantation of the new flowsecure*™* artificial urinary sphincter in the female bladder neck. *Urologia Internationalis*.

[13] Knight SL, Susser J, Greenwell T, Mundy AR, Craggs MD (2006). A new artificial urinary sphincter with conditional occlusion for stress urinary incontinence: preliminary clinical results. *European Urology*.

[14] Alonso Rodriguez D, Fes Ascanio E, Fernandez BL, Vicens VA, Garcia-Montes F Four years experience with the FlowSecure Artificial Urinary Sphincter. Problems and solutions.

[15] Lima SVC, Araújo LAP, Vilar FO, Kummer CL, Lima EC (1996). Combined use of enterocystoplasty and a new type of artificial sphincter in the treatment of urinary incontinence. *Journal of Urology*.

[16] Schiavini JL, Damião R, de Resende Júnior JAD, Dornas MC, Cruz Lima da Costa DS, Barros CB (2010). Treatment of post-prostate surgery urinary incontinence with the periurethral constrictor: a retrospective analysis. *Urology*.

[17] Lima RS, Barros EGC, Souza CA, de Vilar FO, Lima SVC (2011). Periurethral constrictor: late results of the treatment of post prostatectomy urinary incontinence. *International Brazilian Journal of Urology*.

[18] De O Vilar F, Araújo LAP, Lima SVC (2004). Periurethral constrictor in pediatric urology: long-term followup. *Journal of Urology*.

[19] Lima SVC, Araüjo LAP, Vilar PO (1997). Further experience with the periurethral expander: a new type of artificial sphincter. *British Journal of Urology*.

[20] Simone G, Guaglianone S, Papalia R, Leonardo C, Ferriero M, Gallucci M (2009). Periurethral constrictor (Silimed): a new device for treatment of mild urinary incontinence Following radical prostatectomy. *Journal of Urology*.

[21] Gousse AE, Madjar S, Lambert MM, Fishman IJ (2001). Artificial urinary sphincter for post-radical prostatectomy urinary incontinence: long-term subjective results. *Journal of Urology*.

[22] http://zephyr-si.com/zephyr/index.php?lg=en.

[23] Sandul A, Martins FE, Barros P, Lopes TM, Llorens C The ZSI 375 artificial urinary sphincter: a new device for male urinary incontinence.

[24] Malaeb BS, Elliott SP, Lee J, Anderson DW, Timm GW (2011). Novel artificial urinary sphincter in the canine model: the tape mechanical occlusive device. *Urology*.

[25] Brantley Scott F, Bradley WE, Timm GW (1973). Treatment of urinary incontinence by implantable prosthetic sphincter. *Urology*.

[26] Montague DK (1984). Evolution of implanted devices for urinary incontinence. *Cleveland Clinic Quarterly*.

